# The secondary frame in spider orb webs: the detail that makes the difference

**DOI:** 10.1038/srep31265

**Published:** 2016-08-10

**Authors:** Alejandro Soler, Ramón Zaera

**Affiliations:** 1 Universidad Carlos III de Madrid, Department of Continuum Mechanics and Structural Analysis, 28911 Leganés, Madrid, Spain

## Abstract

Spider orb webs are multifunctional structures, the main function of which is to dissipate the kinetic energy of the impacting prey, while minimizing structural damage. There is no single explanation for their remarkable strength and ductility. However, it is clear that topology is decisive in the structural performance upon impact, and the arrangement of the different silk threads in the web must also exert an effect. The aim of this study is to show how a slight variation in the geometry markedly affects the prey-capture ability of spider orb webs. The study is focused on the secondary frame, a thread interposed between radial and primary frame strands, the importance of which has not been examined until now. The simulation of the impact performance of webs using different lengths of the secondary frame clarifies its structural role, which has proven to be decisive. Furthermore, the study explains why secondary frame threads of moderate length, as commonly encountered, enable the capture of prey with higher energy without a marked increase in the volume of silk used.

An orb-weaving spider’s likelihood of survival is influenced by the ability of its web to withstand prey impact with minimum damage and at the lowest manufacturing cost^1^,^2^,^3^,^4^,^5^. This set of requirements has forced spider silk to evolve towards extreme strength and ductility to a degree that is rarely observed among other materials, either natural or manmade^6^,^7^,^8^,^9^,^10^,^11^,^12^,^13^,^14^,^15^,^16^,^17^. However, the superior performance of the orb web as an aerial prey trap is not due merely to the exceptional mechanical properties of the silk, but also to outstanding structural topology^18^. The two factors are closely related, with the arrangement of the threads making the most efficient use of the different silks strands spun by the spider^19^. Indeed, the orb web is typically composed of compliant adhesive spiral threads, the main function of which is prey retention, supported by a scaffold made of strong and rather stiff major ampullate threads (mooring, frame and radii) keeping the sticky silk in place, dissipating the prey energy, and transmitting the impact load to the substrate^20^,^21^ (Fig. 1a).

The primary function of the orb web is to intercept, stop and retain prey long enough for the spider to catch it. The prey impact may challenge the strength of the web, and failure may occur in the spiral sub-structure or in the scaffold. Failure of spirals does not involve major structural damage but may lead to an eventual escape of the prey. Although orb webs show remarkable damage-tolerant features, the failure of scaffold threads may reduce the structural performance to some extent. According to various authors^22^,^23^, spiders consistently repair damage that threatens the integrity of the web, restoring its structural performance and re-establishing the tension in radial threads, thereby mantaining its ability as a prey-trap and sensory system. In particular, spiders readily add new threads when anchors or frames break^23^. In any case, the repair process consumes resources, and the greater the damage the higher the energy expenditure. Therefore, any improvement in the topology of the web that enhances its structural performance and decreases the probability of failure in its scaffold should be considered as beneficial.

The strength of the web depends heavily on the optimal distribution of the silk (a limited and valuable resource for the spider) among the different thread types, and on the appropriate positioning of these threads. Uncovering the strategies that contribute to this requirement in a structure of superior performance, such as the web, not only helps us reach an understanding of how it evolved, but also provides design principles that might apply to other structural systems. Despite the ample research on the mechanical behavior of the orb web, the importance of the *secondary frame* (Fig. 1a) in its structural behavior has been generally overlooked. This part of the scaffold requires a small fraction of the silk compared to spirals and radials, but its contribution to an even distribution of stiffness among the threads is crucial according to the findings presented here.

The authors identified secondary frames (or radials with Y-structure, actually a smaller secondary frame) in orb webs spun by spiders of the family Araneidae, genuses *Araneus* (9 out of 11 webs), *Cyclosa* (8 out of 8 webs), *Zygiella* (13 out of 14 webs) and *Argiope* (4 out of 4 webs), as well as in webs of the family Nephilidae genus *Nephila* (5 out of 5 webs). Secondary frames were also found in webs of genuses *Zilla*, *Micrathena*, *Eriophora*, *Tetragnatha*, *Acusilas*, *Alpaida*, *Gasteracantha*, *Mangora*, *Nuctenea*, *Scoloderus*, and *Wixia* (family Araneidae), and in webs of genus *Anapistula* (family Symphytognathidae), although the authors have only one web picture of each genus at their disposal. Figure 2 shows some representative examples of webs with secondary frames.

In all the above cases spiders systematically avoid: *i*) connection between primary frame and radial threads aligned with the mooring, by interposing secondary frame threads; *ii*) contact between the ends of contiguous segments of the secondary frame. When no secondary frames were found, the spiders avoided connecting radials to the frame near an anchor. In this respect, the authors found a picture^1^ of an *Uloborus diversus* (family Uloboridae) which does not present a secondary frame but, strikingly, the spider avoids radials aligned with the mooring by curving them as they approach the anchor. Similar considerations have been previously stated by Zschokke^20^. This study seeks to provide a rationale for all these observations, based on well-established principles of structural mechanics.

## Results

We report the results that demonstrate the mechanisms behind the beneficial effect of the secondary frame, to highlight its differential effect in web performance and to quantify its benefit. A finite element model was developed to simulate the impact of prey onto an orb web.

**Figure 1:**
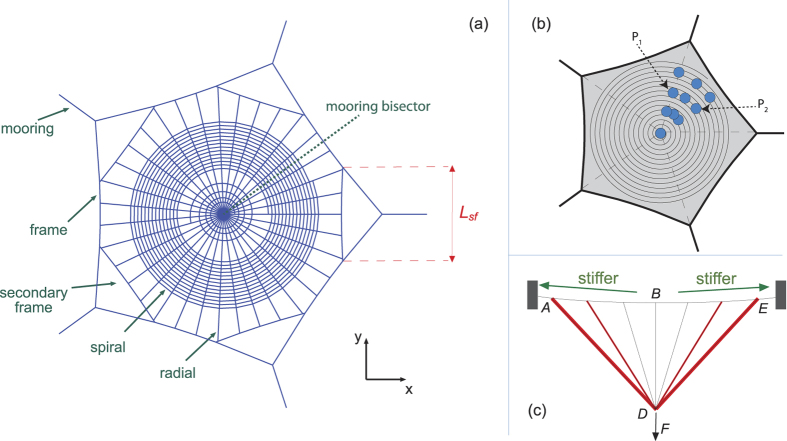
(**a**) Geometry of the reference web before pre-stress; *L*_*sf*_ represents the length of the secondary frame. (**b**) Position of impact points covering half a sector of the web. (**c**) Load path in a set of concurrent threads being pulled towards a cable to which all threads are connected. This structure presents topological similarities to the arrangement of radial threads connected to a segment of the primary or secondary frame in an orb web.

Starting from a reference web geometry, depicted in Fig. 1a, we considered orb webs with different lengths of secondary frame *L*_*sf*_, scaling the length 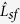 of the reference geometry by a factor 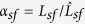 and adopting five different values *α*_*sf*_ = {0, 0.6, 1.0, 1.4, 1.7}. The extreme case *α*_*sf*_ = 1.7 corresponds to the maximum feasible length of the secondary frame, with ends of contiguous threads meeting at the mid-point of the primary frame. This case, as well as the opposite one *α*_*sf*_ = 0 (absence of secondary frame), are not realistic. However, they provide the most valuable results in order to discover why spiders use intermediate lengths, i.e. leaving a certain distance between contiguous connections to the primary frame. In other words, the benefits of using an intermediate length are shown by the analysis of other potential configurations that reveal major structural malfunctions or useless expenditure of silk without any apparent structural benefit. See Methods section for more detailed information on the methodology, and the Supplementary Information for additional technical details.

**Figure 3:**
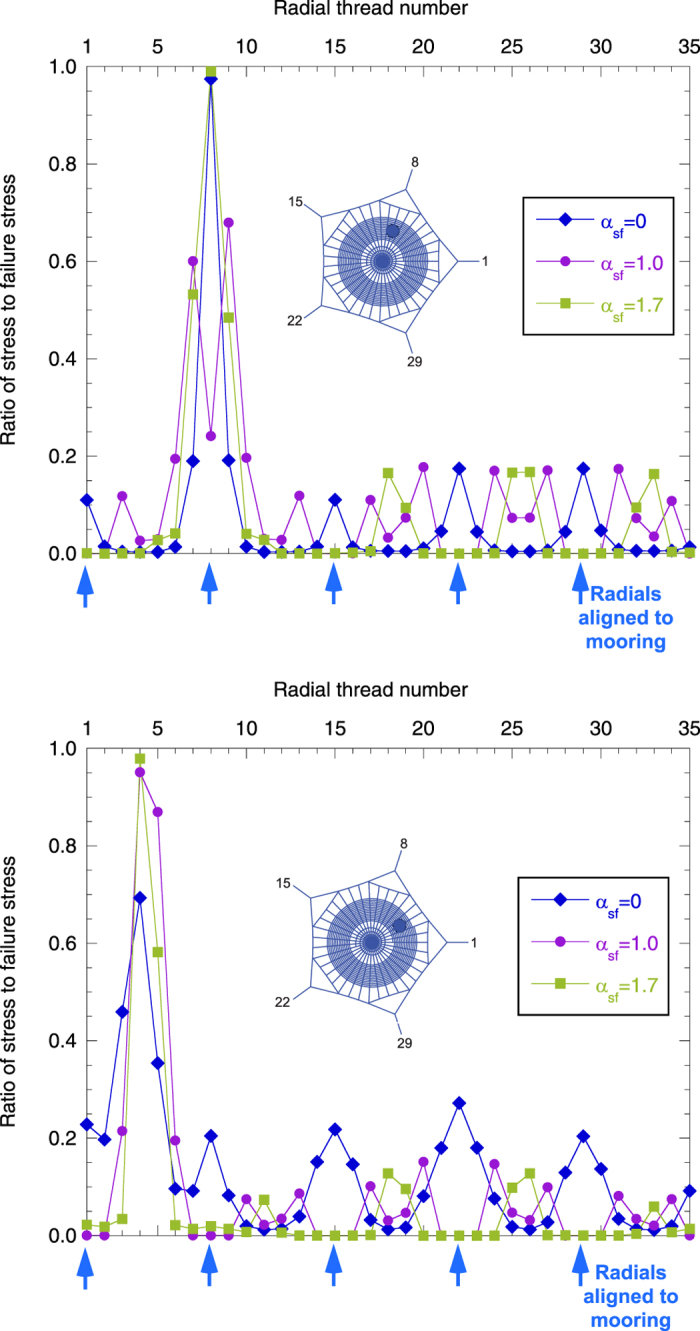
Distribution of stresses in radials at failure, for three different secondary frame lengths *α*_*sf*_ = {0, 1.0, 1.7}. (**a**) impact in a radial aligned with the mooring. (**b**) Impact in a radial aligned with the mooring bisector.

**Figure 4:**
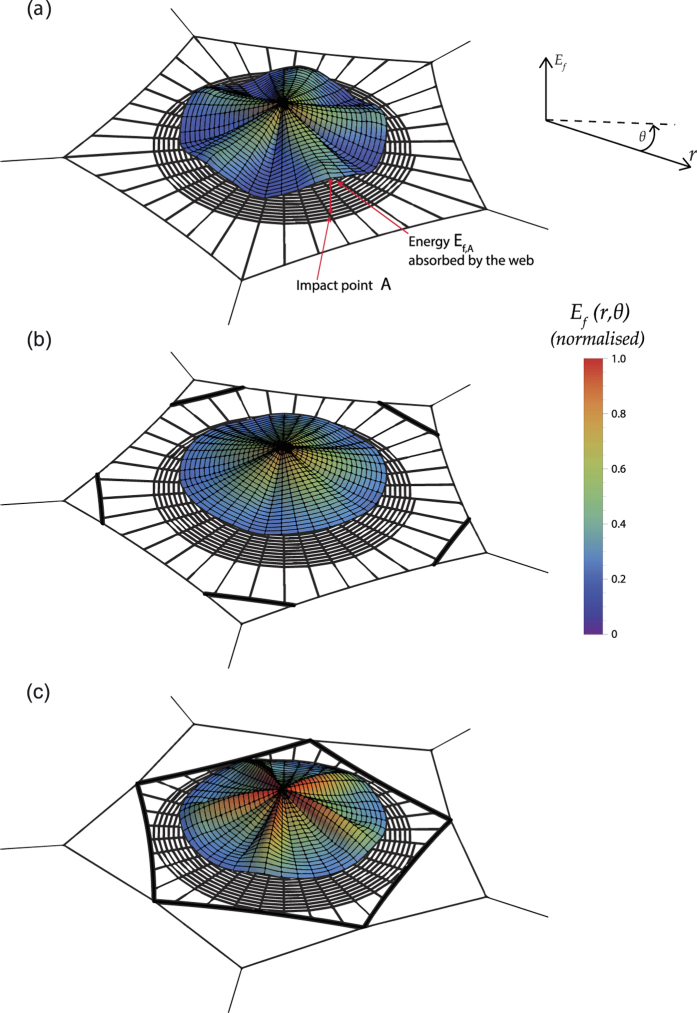
Work exerted by the prey (equivalent to the energy absorbed by the web) up to failure, depending on the contact point between the prey and web. The whole capture area is considered for a potential impact. The continuous energy surface is determined by the interpolation of the values corresponding to the 10 impact points depicted in Fig. 1b. (**a**) *α*_*sf*_ = 0. (**b**) *α*_*sf*_ = 1.0. (**c**) *α*_*sf*_ = 1.7. The values are normalized with the peak value of the energy for the reference case (*E*_*f*_|_*r*=0_ = 1.37 mJ for *α*_*sf*_ = 1.0).

Figure 3 shows the distribution of stresses reached at each radial thread at the failure of the web. Three different lengths of the secondary frame were considered: *α*_*sf*_ = 0 corresponding to an absence of a secondary frame, *α*_*sf*_ = 1.0 corresponding to the reference length of secondary frame, and *α*_*sf*_ = 1.7 corresponding to the maximum possible length of the secondary frame (webs depicted in Fig. 4 show the three different lengths). For this study, the two impact points *P*_1_ and *P*_2_ (Fig. 1b) were considered: the first one on a radial aligned with a mooring, and the second one on a radial aligned with a mooring bisector (Fig. 1a). In both cases, the influence of the secondary frame length in the structural performance of the web is noticeable. Leaving aside the radials directly contacted by the prey (thereby submitted to the peak stress), in the absence of a secondary frame *α*_*sf*_ = 0 the stress is concentrated in the radials aligned with the mooring. Conversely, the stress is concentrated in the radials aligned with the mooring bisector for the maximum length of the secondary frame *α*_*sf*_ = 1.7. Recalling a design criterion commonly accepted in structural mechanics, elements showing a higher stiffness support the main part of the load. Therefore, Fig. 3 suggests that the length of the secondary frame influences the distribution of stiffness among radials. The rationale for this observation and its profound implications in the overall performance of the web under an impact load will be described in the next section.

The ability of the orb web to dissipate the kinetic energy of prey depends largely on the impact point^1^. A suitable way to show which parts of the web are weaker or stronger is by plotting the values of the work exerted by the prey up to failure *E*_*f*_, equivalent to the energy absorbed by the silk structure, as a function of the impact point. The impact zone was noticeably restricted to the capture area, and the continuous function *E*_*f*_(*r*, *θ*) (in polar coordinates) was determined by the interpolation of the values of energy corresponding to the 10 impact points depicted in Fig. 1b. We must emphasize that single impact points on the original, unstretched and undamaged configuration were simulated in all cases (see Methods section). Figure 4 shows the distribution of absorbed energy *E*_*f*_ for three different values of the secondary frame length *α*_*sf*_ = {0, 1.0, 1.7}, with the scale of the vertical axis (energy) being the same for each figure. As an example of the valuable information provided by this figure, it shows that the web with *α*_*sf*_ = 0 impacted at point *A* (Fig. 3a) dissipates the energy *E*_*f*,*A*_. This enables us to visualize which areas are weaker and which are stronger when submitted to a load. Also, the comparison of the different sub-figures makes it possible to identify the effect that variations in the length of the secondary frame exert on: *i*) the distribution of weak/strong areas; *ii*) the average level of energy. This effect will be discussed in detail in the next section.

It should be stressed that the absorbed energies have been calculated using webs submitted to an initial pre-stress state, this being consistent with experimental observations^24^. The energy values derived when pre-stress is disregarded were slightly higher, suggesting that pre-stress is detrimental for energy absorption as the threads are already loaded (therefore closer to failure) before impact. However, pre-stress is required for the proper transmission of sensory information to the spider through thread vibrations^25^, and for keeping webs taut and functional in windy conditions.

## Discussion

Upon prey capture, the orb web has to transmit the contact force from the impact point to the mooring, thereby playing a structural role. An efficient way to visualize how the force is transferred from its application point to the boundaries is by drawing the *load paths*, indicating how load *flows* through the different elements. In cable structures, such as a spider web, the paths can be easily depicted by drawing thicker threads proportionally to the intensity of the transmitted force. No other graphic symbol is required since ideal cables transmit a single internal force type (axial) in a single direction (tension). A representative example of the diagram of load paths in a cable structure is shown in Fig. 1c, where a horizontal cable attached at its ends is being pulled downwards concurrently by different threads. Due to the flexibility of the cable, each joint between the thread and cable moves towards point *D*. However, joints closer to the ends *A* and *E* of the cable require a higher pulling load to be displaced downwards due to the proximity of the stiff clampings. Also, joints closer to the midpoint *B* show a higher flexibility when pulled downwards. Consequently, the force on a specific thread will increase with proximity to points *A* or *E*. In a structure with a strong stiffness mismatch, significantly stiffer elements are commonly prone to premature failure. Therefore, it becomes apparent that the structure in Fig. 1c is not working correctly. Actually, the overall performance will not be significantly altered if the central threads are removed since the load flows through the most taut elements, whereas the most slackest will bear a small part of the external force.

The above study on the performance of the structure depicted in Fig. 1c is central for the discussion of the results presented in this work, due to its topological similarity to the arrangement of radial threads connected to a segment of the primary or secondary frame in an orb web. It provides an understanding of the distribution of stresses among the different radial threads, depicted in Fig. 3. In the absence of a secondary frame *α*_*sf*_ = 0, the stiffest radial-to-frame joints are those corresponding to radial threads connected to the ends of the primary frame threads which are attached to the mooring. In contrast, joints closer to the mid-point of the primary frame are more flexible. Accordingly, the stress increases towards the threads aligned with the mooring. However, regarding the web with the maximum length of the secondary frame *α*_*sf*_ = 1.7, the distribution of stresses is reversed: the stress increases towards the radials aligned with the mooring bisector since now the stiffness increases as we approach the joint between two secondary frame threads. Consequently, both extreme cases show a subset of radial threads with higher probabilities of being damaged. Instead, an intermediate length of the secondary frame (e.g. *α*_*sf*_ = 1.0) evens out the distribution of stiffness among the different radials.

**Figure 5:**
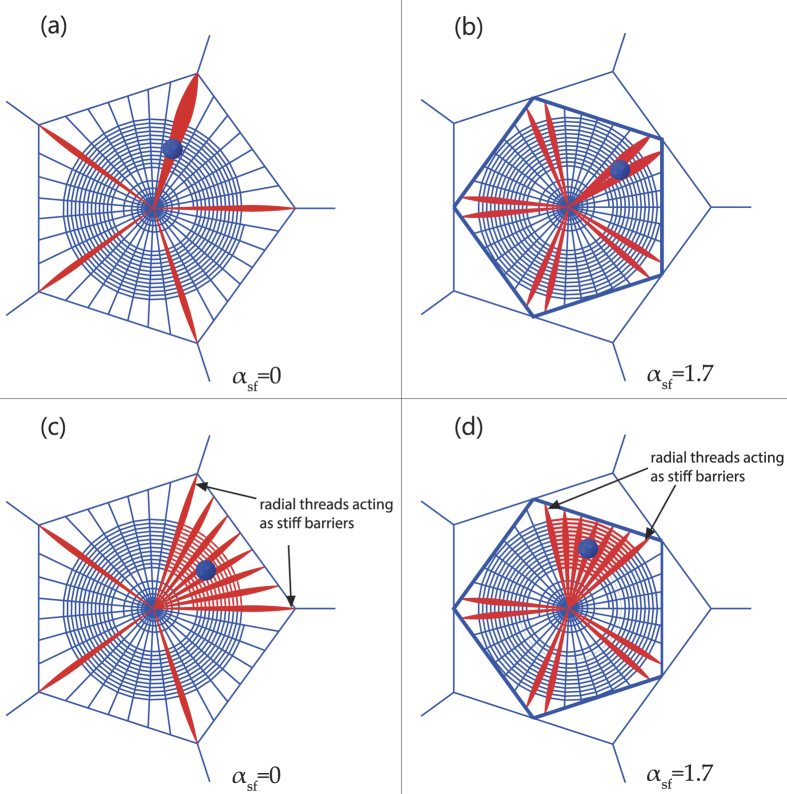
Load paths for different potential impact points, and for extreme cases of the secondary frame length *α*_*sf*_ = 0 and *α*_*sf*_ = 1.7. (**a**,**b**) the impact on stiffer radials; (**c**,**d**) the impact on the bisector or stiffer radials. The load is carried mainly by the stiff radial threads, or confined to the sector limited by them.

As a consequence of the above, several structural malfunctions can be identified in the absence of a secondary frame, or when it is too large. A subset of radial threads is connected to stiff joints, bearing most of the load and preventing others from playing a structural role. Moreover, the stiffer radial threads remain aligned with the reference (initial) plane of the silk structure, facing a fairly normal angle to the load direction (in case of the worst impact event, namely perpendicular to the web). Cable structures perform inefficiently under these conditions, being prone to failure and precluding the absorption of prey energy (Fig. 5a,b). Also, if the impact takes place away from the radial threads connected to stiff joints, these act as a barrier impeding the load from being distributed throughout the entire structure. The stress is then localized in the impacted sector, where the spiral threads are likely to fail (Fig. 5c,d). Another drawback results from the presence of stiff radial threads: the compliance of the whole web and its deformation upon impact decreases. Therefore the potential advantage of energy dissipation by aerodynamic drag, which can play a critical role^26^,^27^,^28^, is largely wasted.

It should also be noted that the distribution of radial stress at failure depicted in Fig. 3 is consistent with findings by Cranford *et al*.^14^. The synergy between the material and the shape of the orb web provides sacrificial elements as a means of avoiding potentially damaging loads, and reducing widespread structural damage. As it can be seen in Fig. 3, the effect of the prey load is localized in the radial threads directly affected by the impact, regardless of the impact location and length of the secondary frame. A few radials take the load while others are well below the silk strength. This leads to a localized failure pattern, allowing the web to remain functional. However, functional does not mean that the ability of the impacted web to arrest a prey remains at the same level. Any degradation, whether from a slight non-recoverable deformation in a thread or from a complete rupture of a radial, causes the web to remain slack and decreases the energy-absorption capacity of the web for future impact events. Moreover, even if spiders are able to effectively repair webs^23^, they expend energy in the restoration process. Therefore, any improvement in the arrangement of silk threads that decreases the probability of damage should be considered beneficial. In this sense, an intermediate length of the secondary frame thread will result in a better impact performance of the web without a marked increase in the volume of silk invested. This latter statement will be further developed below, with an analysis of the energy absorbed by the web and of the probability of failure upon prey capture.

The above analysis on the distribution of stresses among the different threads is now completed by the following analysis focused on energy considerations. As shall be seen below, the two analyses are closely related since the ability of the web to dissipate the impact energy of prey (which may potentially impact on any point of the capture region) is a direct consequence of a balanced distribution of stiffness among its radials. Figure 4 offers a broad perspective of the structural performance of the web, since any possible impact point on the capture zone is considered. First, focusing on the two extreme cases α_sf_ = {0, 1.7}, we see two opposing energy profiles. In the first, corresponding to *α*_*sf*_ = 0 (Fig. 4a), the web provides the minimum capacity of energy absorption if impacted along the radial threads aligned with the mooring. As discussed above, these threads are likely to fail because they are joined to stiff points. In contrast, as the prey impacts on radial threads far from the mooring, the web exhibits higher compliance and stronger behaviour. Therefore, in the absence of a secondary frame, the structural response depends heavily on the impact point. Moreover, the average value of the energy is lower than that of Fig. 4b,c. In general, we find an unreliable performance of the structure as a prey trap.

We now focus on the profile corresponding to the maximum length of the secondary frame *α*_*sf*_ = 1.7 (Fig. 4c). In this case, the distribution of peak values is opposite to that of the previous case. The web provides the maximum capacity of energy absorption if impacted along the radial threads aligned with the mooring. Now these threads are joined to flexible points leading to good performance, whereas radial threads aligned with the mooring bisector are stiffer, leading to a lower dissipation of energy if impacted. Comparing the energy values with those resulting for *α*_*sf*_ = 0, the dispersion is lower and the average is higher, showing the beneficial effects of the secondary frame. However, relatively large differences persist between values at the crests and valleys of the surface.

**Figure 6:**
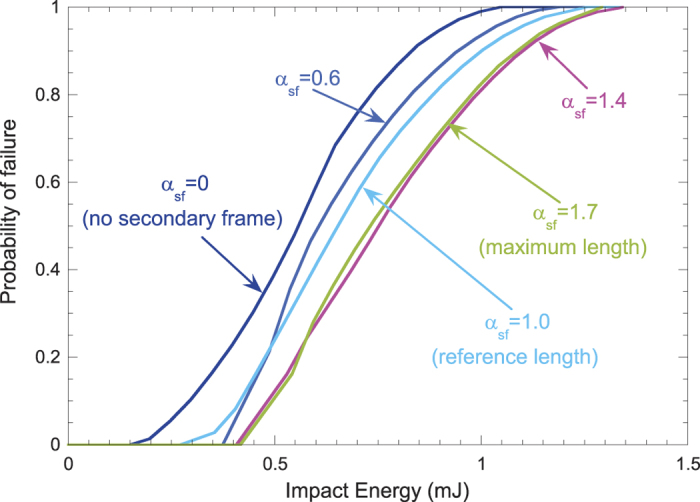
Probability of web failure vs. impact energy for different lengths of the secondary frame.

Figure 4b shows a transitional behavior for an intermediate value *α*_*sf*_ = 1.0, with a greater evenness in the spatial distribution of energies, as it was observed in the distribution of radial stresses, and a more balanced performance. In any case, it is useful to *quantify* this performance and compare it to results corresponding to other lengths of the secondary frame. Assuming that any point in the capture zone has the same probability of being struck by the insect, and using the function *E*_*f*_(*r*, *θ*) providing the distribution of absorbed energies, we can calculate the probability of failure of the web for a given value of the kinetic energy of the prey (see Methods section). Figure 6 shows the corresponding results for the five different lengths of the secondary frame considered in the analysis. The web without a secondary frame (*α*_*sf*_ = 0) shows the worst performance, with the highest probability of being damaged for a given value of the impact energy. As soon as the secondary frame is added (*α*_*sf*_ = 0.6) the improvement is noticeable, highlighting again the importance of this thread, and the benefit increases with its length.

Notably, long secondary frame threads (*α*_*sf*_ > 1.4) do not result in an additional gain since, as stated above, some radials start to show stiffer behavior. Moreover, examining the manufacturing cost, we find that a web with a longer secondary frame does come at an extra silk expenditure: the volume invested in the orb web increases roughly by 6% from *α*_*sf*_ = 0 to *α*_*sf*_ = 1.7. Therefore, beyond a certain length it is not profitable for the spider to spin webs with longer segments of secondary frame, as the structural benefits are not appreciable and, additionally, the capture area is diminished. The observation of real orb webs provides evidence that spiders systematically avoid radials connected to the mooring by interposing a secondary frame thread, but the ends of these threads are never placed very close to each other. The finding of an optimal value of *α*_*sf*_ confirms the benefit of using a moderate length of the secondary frame. The specific value found here is certainly related to the particular conditions of the current work. In any case, this analysis is valuable for understanding how spiders utilize a limited amount of silk to create architecturally superior structures.

The early stage of orb web construction, when anchor, frame and initial radii are laid, does not follow a fixed behavioral pattern^29^,^30^. Rather, the spider reacts in a flexible manner to adapt to a highly variable environment. This means that the spider places, moves and takes down threads until the proto-hub with few proto-radii emerges. Later on, some segments of these radials become part of the frame or of the mooring, as the spider attaches new threads to them and shapes the final scaffold of the web. Therefore, the placement of initial radials, frame and mooring does not follow a clear order of precedence. In any case, the structural performance of the orb web is defined by its final topology, that is, the way in which the silk threads are connected to each other, thereby leading to outstanding performance. And this is where the importance of the construction process emerges: the spider succeeds in avoiding radials connected too close to the anchors, in order to reach an even distribution of stiffnesses among radials that would eventually lead to premature failure. The secondary frame ensures a highly efficient solution at a low silk cost, without the need of curving radials or enlarging the distance between neighbouring radii around the mooring threads.

There is a strong trend in nature to make the maximum use of finite resources; saving operational energy allows an organism to allocate more energy to reproduction. According to this study, the use of the secondary frame, a feature of the web that has been commonly disregarded, allows the spider to reach higher structural efficiency. Certainly the web is a multi-functional system and other factors may influence its geometry. In any case, uncoupling each of these factors and treating them separately is a suitable methodology to improve our overall understanding of the remarkable performance of the orb web, and to make significant advances into creating bioinspired structures. It becomes clear that in the strong evolutionary contrast between topological changes in webs that enhance prey interception and retention and those that reduce energetic costs, nature finds major opportunities for improvement through subtle –and seemingly irrelevant– details.

## Methods

This section describes the methodology devised to show the influence of the secondary frame length in the structural performance of the web, and the key features of the model. See the Supplementary Information for additional technical details.

### Web geometry

The reference orb web is similar to that used in Zaera *et al*.^28^. This geometry was defined by approximating the characteristics of the web built by *Araneus diadematus*^27^, in agreement with the models proposed by other authors^26^,^31^. The primary frame is a regular pentagon, the distance from the center to each vertex being 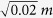. The capture zone is an Archimedean spiral with an area of 0.033 *m*^2^. Each of the five sectors contains seven radial threads. Figure 1a shows the geometry of the *reference web*, with a length of the secondary frame 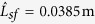 (see Supplementary Information 1 for the complete definition of the parameters of the reference web).

### Constitutive model of the silk

The mechanical behaviour of the silk is captured by a microstructure-based continuum model proposed by De Tomassi *et al*.^32^. Some modifications have been included for better performance (see Supplementary Information 3). According to this model, a silk protein macromolecule is composed of two phases: a soft phase with an entropic elastic behavior acting as the amorphous regions, and a hard phase, which represents the behavior of the *β*-sheet crystals. Therefore, a unit volume of silk thread is composed of a mixture of soft fraction *α*_*s*_ and hard fraction *α*_*h*_ (*α*_*s*_ + *α*_*h*_ = 1). When the hard phase is stretched to its limit (emulating the breakage of a hydrogen bond), it undergoes a transition to a soft phase. Whereas the soft fraction is always active, the hard phase presents an active and an inactive fraction. The hard phase is activated following a strain-dependence probability distribution 
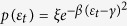
, where *ε*_*t*_ is the threshold strain from which the transition starts, and *β*, *γ* and *ξ* are parameters related to the material. The stress in a silk thread is given by the contribution of both the soft and hard phases (see Supplementary Information 3).

The model by De Tomassi *et al*.^32^ considers the self-healing effect observed by Denny^33^ in a possible reloading of the thread after having being relaxed for several min, or after exposure to heat. This permits the silk to recover its original properties before a load-unload cycle. Owing to the short duration of the impact problem studied (around 250 milliseconds), this effect has been disregarded for subsequent load cycles due to the high frequency vibration of the web upon impact, and the conversion of hard phase to soft phase cannot be reversed.

### Simulation of impact

Owing to the variability found among potential prey species, a representative shape and impact velocity was used: a 20 mm diameter rigid sphere approaching perpendicularly at 2 m/s. The prey was modeled as a rigid surface given the lack of values of stiffness available in the literature. The velocity was kept constant over time in order to force failure to occur in any of the scaffold threads (radials, frame or mooring). While this type of impact does not represent a real one (the prey decelerates as a result of contact with the web), it permits the calculation of the energy absorbed by the web until failure, as a measure of the ability of the web to stop prey in terms of its kinetic energy. Then, the following key variables were measured: *i*) stress reached by each radial thread at failure, *ii*) work exerted by the rigid sphere on the web up to failure, equivalent to the energy absorbed by the silk structure. The first variable permits an evaluation of the distribution of the impact load among the different threads, whereas the second represents the ability of the web to stop prey at a given kinetic energy.

### Finite-element model

The simulations exhibit the main sources of non-linearity in the mechanics of solids: material behaviour, finite deformations, and contact, added to aerodynamic drag. These features, combined with the short duration of the event, make the use of an explicit finite-element code recommendable for solving the set of non-linear equations. The explicit solver of the finite-element code ABAQUS v.6.14-2^34^ was used for this purpose. The spider orb web was modeled using a parametrized script defining finite-element mesh, loads, and boundary conditions. The silk threads were represented by two-node linear displacement truss elements, defined through a user subroutine which considers the silk constitutive model and the aerodynamic drag (see Supplementary Information 5). The cable-like behavior of the thread was forced by discretizing each segment of the web with at least two elements, introducing at least an intermediate hinge along each segment to capture the low bending stiffness of such a slender thread. In this way, just a slight compressive force induced the misalignment of the elements. The aerodynamic force follows a proper description of the drag force applied to a cylinder, considering the relation between the drag coefficient and the Reynolds number^35^,^36^. The reference configuration of the orb web was assumed to be pre-stressed, considering characteristic tension values found elsewhere^24^.

### Evaluation of the overall performance of the web under impact

A proper evaluation of the performance of the web under prey impact requires the analysis of any potential contact point, given that its structural response depends on the point of impact^1^. To do this, the following methodology was developed in order to measure the influence of the secondary frame length in the overall efficiency of the web as a prey trap. For each of these five geometries, 10 different impact points evenly distributed over half a sector of the capture area were selected (Fig. 1b). Each impact was simulated considering the original, unstretched, and undamaged web. Using the web symmetries, and interpolating results for points in the neighborhood of this 10 reference points, we can calculate the energy absorbed by the web for any impact point in the capture area (Fig. 4).

Moreover, the probability of web failure for a given impact energy (Fig. 6) was calculated using the following approach. Starting from the interpolated function of failure energy in polar coordinates *E*_*f*_(*r*, *θ*), we define a *perforation function* as


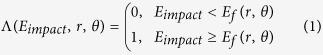


giving zero output if the impact energy *E*_*impact*_ at a certain point is below that leading to failure, and unit output if not. By assigning equal probabilities of impact to any point of the capture zone, we can define the probability of web failure for a given value of the impact energy


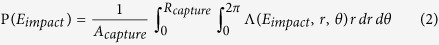


where *R*_*capture*_ and *A*_*capture*_ are, respectively, the external radius and the area of the capture zone.

## Additional Information

**How to cite this article**: Soler, A. and Zaera, R. The secondary frame in spider orb webs: the detail that makes the difference. *Sci. Rep*. **6**, 31265; doi: 10.1038/srep31265 (2016).

## Supplementary Material

Supplementary Information

## Figures and Tables

**Figure 2:**
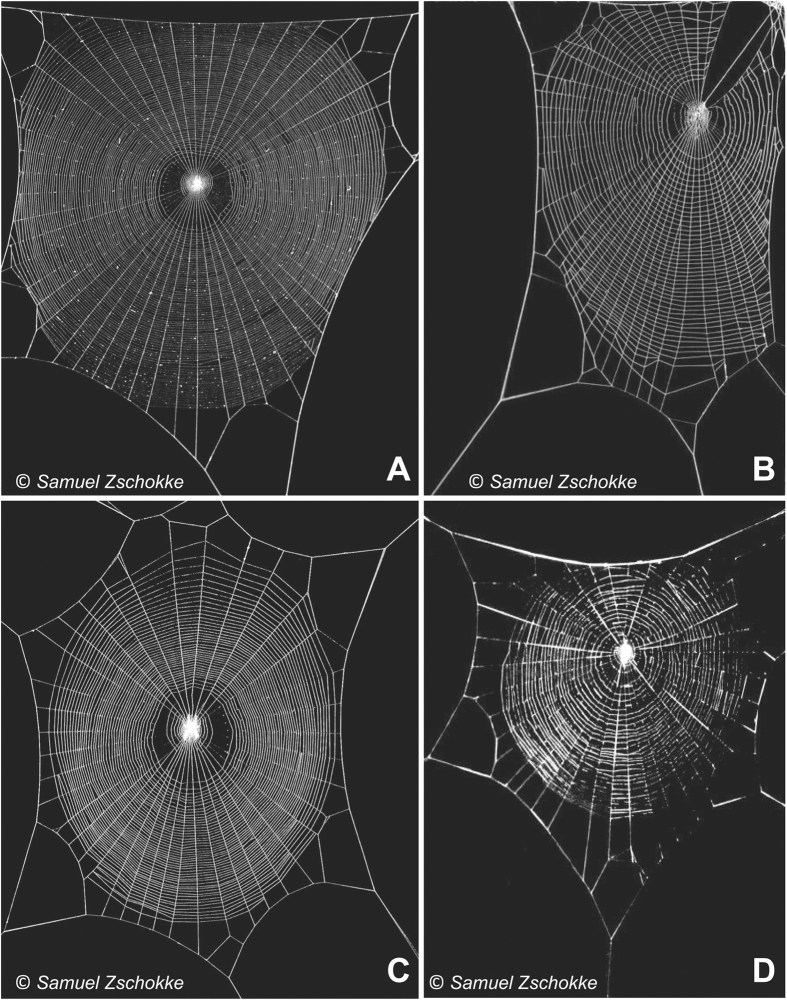
Pictures of orb webs constructed by different spider species. (**A**) Zilla diodia. (**B**) Zygiella x-notata. (**C**) Araneus diadematus. (**D**) Cyclosa oculata. Secondary frames can be seen in (**A**) to (**D**). Radials with Y-structure can be seen in (**C**). Spiders avoid connecting radials to the frame near an anchor, and close proximity between the ends of contiguous secondary frame threads. Courtesy of Dr. Samuel Zschokke (University of Basel).
