# Supplementary material for: The secondary frame in spider orb webs: the detail that makes the difference
Source: Sci Rep. 2016 Aug 10;6:31265. doi: 10.1038/srep31265 (PMC4978998; doi:10.1038/srep31265)
Supplement: Supplementary Information [file srep31265-s1.pdf]

Supplementary Information

The secondary frame in spider orb webs. The detail that makes the difference

Alejandro Soler[1], **Ramón Zaera**[1,*]

[1]Universidad Carlos III de Madrid, Department of Continuum Mechanics and Structural Analysis, 28911 Leganés, Madrid, Spain
[*]ramon.zaera@uc3m.es

Supplementary Information 1

Geometry of the orb web

The orb web geometry is defined by a regular convex polygon of N_s sides and a radius R_p. In each vertex there is a mooring thread with length L_a. The secondary frame is defined by its length L_{sf}. The capture zone is made up of two different threads: N_r radial threads equally separated, and by an archimedean spiral defined by an initial and final radii, R_{c1} and R_{c2} respectively, and by the distance between successive turnings of the spiral C_c. At the centre of the orb web there is a hub logarithmic spiral defined by its length L_h and its initial and final radius R_{h1} and R_{h2} (Supplementary Fig. 1). Supplementary Table 1 has the parameters used in this work to model the *reference* web, identified by a hat $\hat{}$, where changes have been introduced exclusively in the length of the secondary frame threads.

This reference web was defined approximating the geometric characteristics studied elsewhere, and is similar to that considered in the web models presented by other authors[1–7] . The use of a particular geometry prevents the analysis of the features of webs built by specific spiders, given the variability of geometries among species (spider webs exhibit considerable intraspecific variability in various aspects), but it has been considered by the authors as a proper methodology to provide a view on the mechanical behaviour of orb-webs.

Parameter	Symbol	Units	Value
no. of sides	$\hat{N}_s$	-	5
no. of radial threads	$\hat{N}_r$	-	35
radius of polygon	$\hat{R}_p$	mm	141.4
length of mooring threads	$\hat{L}_a$	mm	40.0
length of sec. frame threads	$\hat{L}_{sf}$	mm	86.6
initial radius of hub spiral	$\hat{R}_{h1}$	mm	10
final radius of hub spiral	$\hat{R}_{h2}$	mm	30.0
initial radius of capture spiral	$\hat{R}_{c1}$	mm	40.0
final radius of capture spiral	$\hat{R}_{c2}$	mm	92.0
length of logarithmic spiral	$\hat{L}_h$	mm	400
separation distance of archimedean spiral	$\hat{C}_c$	mm	4.0
mooring, primary and secondary frame threads diameter	$\hat{\phi}_f$	μm	5
radial thread diameter	$\hat{\phi}_r$	μm	3.5
spiral thread diameter	$\hat{\phi}_s$	μm	2.3

Supplementary Table 1. Values of the parameters defining the *reference* geometry (Supplementary Fig. 1), approximating the geometric characteristics studied by Vollrath et al.[8]

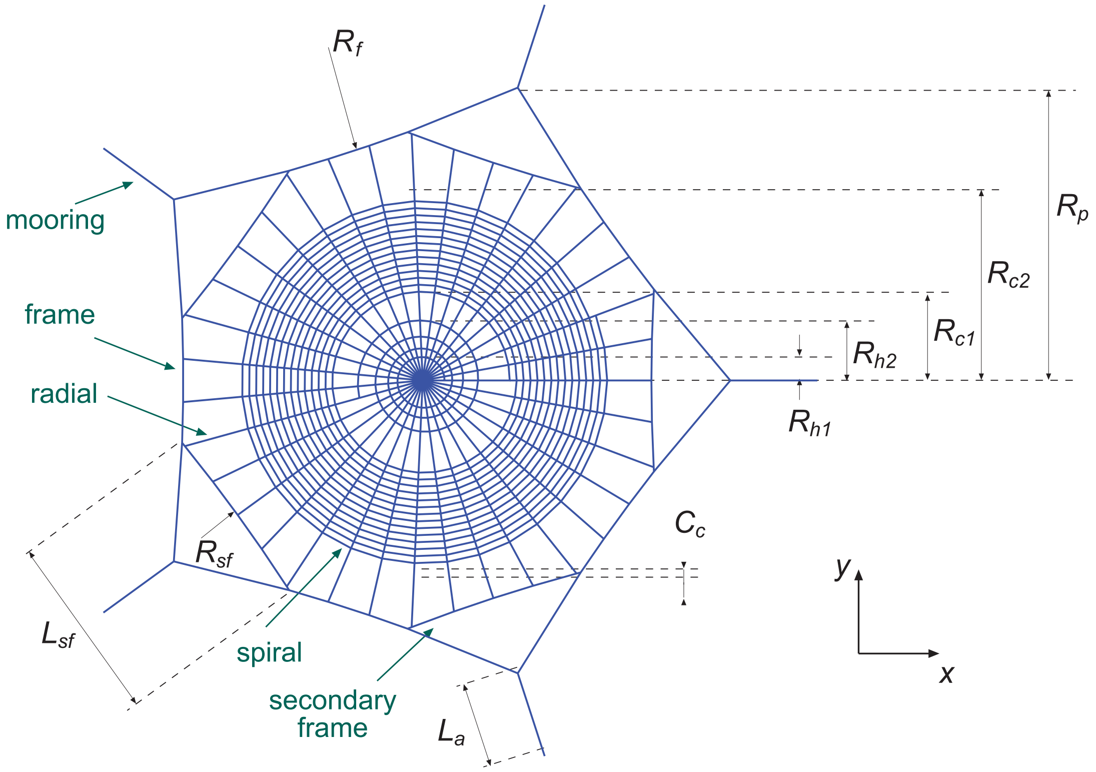

Supplementary Figure 1. Reference orb-web geometry.

Supplementary Information 2

Stress, strain and stretch

As a measure of strain we used the Hencky or true strain (Supplementary Eq. 1), thus the logarithm of the stretch λ defined as the ratio between the deformed length dx and the initial length dX of the thread. This non-linear measure of strain is suitable for large deformation phenomena

$$\varepsilon = ln\lambda \tag{1}$$

By virtue of the hypothesis of volume constancy upon deformation[?, 9, 10] , the initial cross section area A_0 and the stretch, is used to determine the current (deformed) cross section area of the thread

$$A = \frac{A_0}{\lambda} \tag{2}$$

The Cauchy or true stress σ is used, given by the ratio between the force F in a thread and the current area of the cross section.

$$\sigma = \frac{F}{A} \tag{3}$$

Supplementary Information 3

Silk model

A continuum model, based on the micro-structure of the silk, has been considered. This model was proposed by De Tomassi et al,[11] and the authors of the current works have introduced some suitable changes.

The silk is composed by two fractions of soft amorphous α_s and hard crystalline material α_h. Thus, both fraction fulfil the condition

$$\alpha_s + \alpha_h = 1 \tag{4}$$

Adopting the rule of mixtures, the stress in the silk is obtained as

$$\sigma = \alpha_s \sigma_s + \alpha_h \sigma_h \tag{5}$$

The soft phase is active at any value of the strain, contributing with a stress given by

$$\sigma_s(\varepsilon, \varepsilon_c(\alpha_s)) = E_s \left(\frac{1}{4} \left(1 - \frac{\varepsilon}{\varepsilon_c(\alpha_s)} \right)^{-2} - \frac{1}{4} + \frac{\varepsilon}{\varepsilon_c(\alpha_s)} \right) \tag{6}$$

where E_s is the elastic modulus of the soft phase and ε_c acts as a limit strain, which depends on the amount of soft material α_s.

The stress in the hard phase is defined by

$$\sigma_h(\varepsilon) = \begin{cases} 0, & \text{if } \varepsilon \leq \varepsilon_a, \\ E_h(\varepsilon - \varepsilon_a), & \text{if } \varepsilon_a < \varepsilon < \varepsilon_t, \\ \sigma_s(\varepsilon, \varepsilon_c), & \text{if } \varepsilon \geq \varepsilon_t, \end{cases} \tag{7}$$

E_h being the hard phase elastic modulus, ε_a the hard phase activation threshold, and ε_t the transition strain. Thus, the Supplementary Eq. (7) represents the three possible states of the hard phase: inactive for $\varepsilon \leq \varepsilon_a$, active for $\varepsilon_a < \varepsilon < \varepsilon_t$, and transitioned to soft for $\varepsilon \geq \varepsilon_t$. The crystals of the hard fraction have different sizes, and their activation and transition strain ε_a and ε_t varies from one crystal to another. De Tomassi et al[11] assumed in their model that these strains also varies, but not the difference between both, called δ

$$\delta = \varepsilon_t - \varepsilon_a, \tag{8}$$

The soft transition threshold follows a Gaussian probability distribution,

$$p(\varepsilon_t) = \xi e^{-\beta(\varepsilon_t - \gamma)^2} \tag{9}$$

The integration of Supplementary Eq. (9) between 0 and ε which gives the amount of soft fraction transitioned in the material. ξ, β, and γ are material parameters. However, while β and γ are calibrated to the experimental stress-strain curves, the parameter ξ is calculated by imposing

$$\int_0^\infty p(\varepsilon_t) d\varepsilon_t = 1 - \alpha_0 \tag{10}$$

where α_0 is the initial soft phase present in the material. Previous equation introduce a modification in the modal proposed by De Tomassi et al.[11] Thus, the amount of soft material is defined by

$$\alpha_s(\varepsilon_M) = \alpha_0 + \int_0^{\varepsilon_M} p(\varepsilon_t) d\varepsilon_t \tag{11}$$

Here ε_M represents the maximum strain reached in the thread. With the proposed modification, the total amount of soft fraction (initial+transitioned) cannot be greater than one for large values of strain.

The stress-strain relation is given by the contribution of both, soft and hard phases, given by Eq. (12).

$$\sigma(\varepsilon, \varepsilon_M) = \alpha_s \sigma_s + \alpha_h \sigma_h = \alpha_s(\varepsilon_M) \sigma_s(\varepsilon, \varepsilon_c(\alpha)) + E_h \left(\int_{\varepsilon_M}^{\delta} \varepsilon p(\varepsilon_t) d\varepsilon_t + \int_{max(\varepsilon_M, \delta)}^{\varepsilon + \delta} (\varepsilon - (\varepsilon_t - \delta)) p(\varepsilon_t) d\varepsilon_t \right) \tag{12}$$

The first integral takes into account the hard material with activation $\varepsilon_t < \delta$, $\varepsilon_a = 0$ is assumed. The second integral represents the hard material with $\varepsilon_t > \delta$ (integrals whose lower limit is larger than the upper limit are zeroed). Note that when ε equals the maximum strain reached, we are in a loading path ($\varepsilon = \varepsilon_M$), and in unload-reload path for $\varepsilon < \varepsilon_M$. The previous

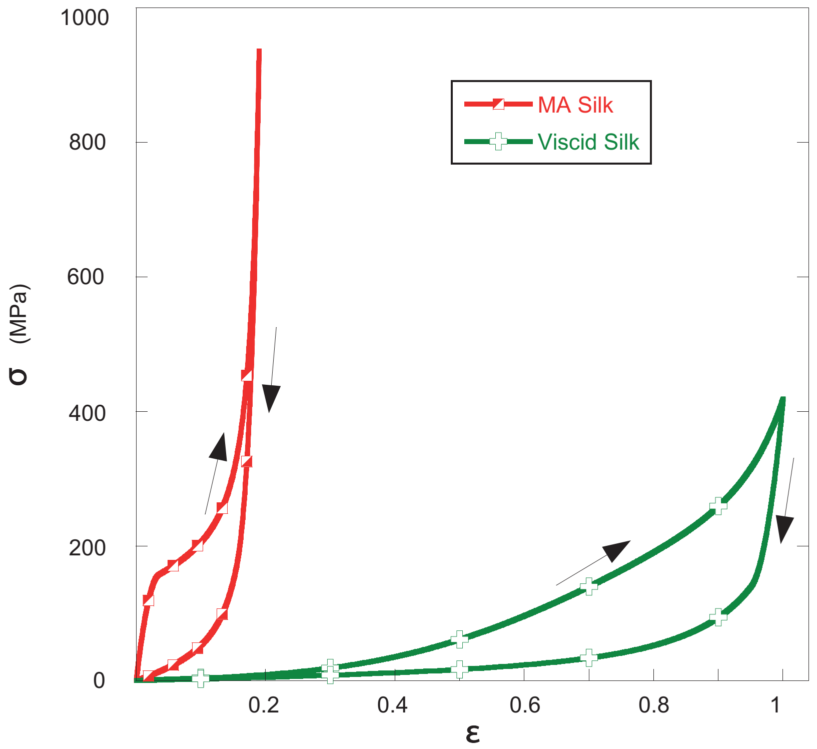

Supplementary Figure 2. Loading and unloading behaviour of MA and viscid silks.

	Soft Elastic Modulus, E_s (MPa)	ε_c -	α_0 -	Hard Elastic Modulus, E_h (GPa)	$p(\varepsilon_t)$ -	δ -	Failure strain ε_{fail} -	Density (kg/m^3)
MA silk	160	$0.025+0.195\alpha_s^{0.006}$	0.15	313	$1.0326e^{-3(\varepsilon_t-0.35)^2}$	0.035	0.19	1098
Viscid silk	35	$0.15+1.05\alpha_s^{0.05}$	0.15	120	$1.0732e^{-5(\varepsilon_t-1)^2}$	0.05	1	1098

Supplementary Table 2. Material parameters defining the stress–strain behaviour for MA and viscid silk (see Supplementary Fig. 2).

expression implies a modification in the model proposed by De Tomassi et al.[11] by using the same integral for the load and for the unload stage, and taking into account when the maximum strain reached ε_M is lower than δ. Considering this condition permits a proper characterization of the mechanical behaviour of the silk in the range of low strains for a dynamic analysis with high frequency oscillations. The changes introduced in the model allow to describe the stress-strain behaviour with a single equation rather than the three used in the original model by De Tomassi et al.[11]

We defined two different silks for the whole web. The mooring, primary frame, secondary frame and radial threads are composed of major ampullate silk (MA silk). On the other hand the spiral threads contains viscid silks. The parameters defining the mechanical behaviour of each type of silk are given in the Supplementary Table 2, leading to the stress–strain shown in the Supplementary Fig. 2.

Supplementary Information 4

Aerodynamic Drag

Silks threads has been idealized as smooth cylinders of diameter ϕ, thus the air drag force per unit length is given by

$$\Psi_D = -\frac{1}{2} C_D \rho_a \phi |\mathbf{v}_n|^2 \mathbf{u}_n \tag{13}$$

where $\mathbf{v}_n$ is the normal component of the thread velocity relative to the surrounding air, $\vec{u}_n$ the unit vector in the direction of $\mathbf{v}_n$ and ρ_a is the air density ($\rho_a = 1.225$ kg/m^3 for air at 300 K and atmospheric pressure). The minus sign means that the force has the opposite direction to that of the normal velocity $\mathbf{v}_n$. C_D is the drag coefficient, defined as dependent on the Reynolds number.[12] In the cases studied here, the Reynolds number is lower than unit and the flow regime is laminar[13,14] . Thus, the relation between C_D and Re is assumed to follow the expression[15,16]

$$C_D = B \cdot Re^{-m} \tag{14}$$

The parameters $B = 12.18$ and $m = 0.629$ are fitted to the experimental results reported by[17] . Hence, combining Supplementary Eqs. (13) and (14) the drag force applied to a silk thread is

$$\Psi_D = -\frac{B}{2} \kappa^m \rho_a \phi^{1-m} |\mathbf{v}_n|^{2-m} \mathbf{u}_n, \tag{15}$$

where κ is the kinematic viscosity ($\kappa = 16 \cdot 10^{-6}$ m^2/s for air at 300 K and atmospheric pressure).

Supplementary Information 5

Truss finite element implementation

The explicit solver of the commercial code ABAQUS 6.14-2 has been used for the simulations. The threads are meshed with two-node linear displacement truss elements, which has provided satisfactory results in previous studies.[13] The element has been implemented in a user subroutine, defining the behaviour in large deformations and rotations[?] .

In the frame of the finite element method, the work done by the internal forces (stresses in the silk) and external forces (aerodynamic drag and contact forces) in the D'Alambert principle can be expressed in terms of internal and external equivalent nodal forces. Linear shape functions are used to relate the displacement field in the element to the corresponding values in the nodes. A co-rotational formulation of the element has been used, thus, a local Cartesian coordinate system $\{x, y, z\}$ rotates with the element, the component x is parallel to the element length, and the components y and z are orthogonal to the element direction. Once the internal and external forces have been calculated, a transformation matrix relates local to global Cartesian coordinate system $\{X, Y, Z\}$.

The internal forces are described in the Supplementary Eq. (12). The stress is constant inside the element due to linear shape functions. In a co-rotational formulation and for a truss element, internal forces are aligned to direction x.

The aerodynamic drag is a velocity-dependent distributed load defined by Supplementary Eq. (15). The velocity field inside the element can be related to the shape function and the nodal velocities. The aerodynamic drag is applied only to the components of the velocity orthogonal to the element length. Thus, in a co-rotational formulation it means only two components for each node. Under the same hypothesis used for the internal, the equivalent nodal external forces in the local system are obtained, integrating the aerodynamic drag load through the element length (integral which has closed-form solution).

References

1. Lin, L. H. & Sobek, W. Structural hierarchy in spider webs and spiderweb-type systems. *Struct. Eng.* **76**, 59–64 (1998).
2. Ko, F. K. & Jovicic, J. Modeling of mechanical properties and structural design of spider web. *Biomacromolecules* **5**, 780–785 (2004).
3. Alam, M. S. & Jenkins, C. H. Damage tolerance in naturally compliant structures. *Int. J. Damage Mech.* **14**, 365–384 (2005).
4. Alam, M. S., Wahab, M. A. & Jenkins, C. H. Mechanics in naturally compliant structures. *Mech. Mater.* **39**, 145–160 (2007).

5. Tarakanova, A. & Buehler, M. J. A materiomics approach to spider silk: Protein molecules to webs. *JOM* doi:10.1007/s11837–012–0250–3 (2012).

6. Tarakanova, A. & Buehler, M. J. The role of capture spiral silk properties in the diversification of orb webs. *J. R. Soc. Interface* **9**, 3240–3248 (2012).

7. Cranford, S. W., Tarakanova, A., Pugno, N. M. & Buehler, M. J. Nonlinear material behaviour of spider silk yields robust webs. *Nature* **482**, 72–76 (2012).

8. Vollrath, F., Downes, M. & Krackow, S. Design variability in web geometry of an orb-weaving spider. *Physiol. Behav.* **62**, 735–743 (1997).

9. Guinea, G. V., Pérez-Rigueiro, J., Plaza, G. R. & Elices, M. Volume constancy during stretching of spider silk. *Biomacromolecules* **7**, 2173–2177 (2006).

10. Simulia. *ABAQUS/Explicit User's Manual, v. 6.14.2* (Dassault Systèmes, Providence, USA, 2014).

11. Tommasi, D. D., Puglisi, G. & Saccomandi, G. Damage, self-healing, and hysteresis in spider silks. *Biophys. J.* **92**, 1941–1948 (2010).

12. Tritton, D. J. *Physical Fluid Dynamics* (Oxford University Press, England, 1988).

13. Zaera, R., Soler, A. & Teus, J. Uncovering changes in spider orb-web topology owing to aerodynamic effects. *J. R. Soc. Interface* **11**, 20140484 (2014).

14. Craig, C. L. The ecological and evolutionary interdependence between architecture and web silk spun by orb web weaving spiders. *Biol. J. Linn. Soc* **30**, 135–162 (1987).

15. Oseen, C. W. Über die stokessche formel und über eine vervandte aufgabe in der hydrodynamik. *Ark. Mat. Astr. Fys.* **6**, 175 (1910).

16. Lamb, H. *Hydrodynamics* (Cambridge University Press, Cambridge, 1932).

17. Tritton, D. J. Experiments on the flow past a circular cylinder at low reynolds number. *J. Fluid Mech.* **37**, 574–567 (1959).
